# Combining forces to improve simulation-based practices for Emergency Preparedness and Disaster Responses

**DOI:** 10.1186/s41077-025-00330-w

**Published:** 2025-02-11

**Authors:** Guillaume Alinier, Linda Sonesson

**Affiliations:** 1 Hamad Medical Corporation Ambulance Service, Medical City, Doha, Qatar; 2 School of Allied Health Professions, Midwifery and Social Work, University of Hertfordshire, College Lane, Hatfield, AL10 9AB UK; 3 Weill Cornell Medicine – Qatar, Education City, Doha, Qatar; 4 Faculty of Health and Life Sciences, Northumbria University, Coach Lane Campus, Benton, Newcastle Upon Tyne, NE7 7XA UK; 5 Florence Nightingale Faculty, King’s College London, 57 Waterloo Rd, London, SE1 8WA UK

Simulation has gained tremendous momentum since the turn of the twenty first century, with a greater focus on the quality of education and training provided to healthcare professionals [1, 2]. Not only has simulation proven to be useful to help individual clinicians acquire skills, it is also now commonly used to train teams to work together, as well as to test systems and processes in diverse domains, including healthcare [3–5]. Simulation is also increasingly used in the healthcare context to help identify latent safety threats, which can, in turn, lead to system improvements, such as enhanced patient safety and higher system reliability [6]. Healthcare has experienced major disruptive changes in response to the COVID-19 pandemic, and both emerging and ongoing conflicts, and technology has been rapidly deployed to maintain teaching and learning, even in austere environments [7–9]. While the future of healthcare education and simulation is still uncertain, the use of technology is highly likely to be an essential component of upcoming transformative changes [10].

This article reports the perspectives of two academics with diverse and complementary experiences in the field of simulation, and advocates for greater collaboration among those involved in organizing emergency preparedness and disaster response (EPDR) in their respective settings. “Combining forces” and sharing knowledge leads to cross-fertilization and sharing of experiences and practices. This, in turn, could contribute to achieving significant improvements and efficiencies, in terms of time spent organizing such types of activities, resource utilization, educational effectiveness, and system impact.

Simulation-based training and education, in its various modalities [11], can help create realistic disaster and mass-casualty situations that provide beneficial learning opportunities within the safety of a controlled environment [12]. However, a qualitative study involving disaster management experts reported that although simulation-based training is the best approach for training, it is not occurring often enough [13]. The consensus of this study, in the context of chemical, biological, radiological, and nuclear incidents, is that there should be greater international training collaboration, but such training is often limited due to geopolitical issues, especially in the Middle East and North African region.

With the ever-present risk of natural disasters, industrial accidents, conflicts, and terrorist attacks, EPDR planning can minimize the effect of such calamities [14]. Closely linked to the principles of Crisis Resource Management (CRM), EPDR cannot be left to chance, nor should it be improvised at the last minute [15]. It requires careful planning and multiagency collaboration, and needs to incorporate training at various levels to ensure appropriate readiness for different potential scenarios. This is where simulation can play such an important role [16]. Emergency preparedness requires planning, equipment acquisition, a written plan, and training [14]. Most of these should be ongoing processes to maintain emergency preparedness at an individual and team level.

Such recommendations are not new. Indeed, diverse groups of individuals specializing in facilitating training for EPDR exist around the globe. However, they are often working in their specific domains, perhaps doing very similar things, yet not sharing their practice or the knowledge generated from their work. They often unknowingly work in silos, according to their preferred simulation modality or specific needs, and are not aware of educational research and evidence-based standards others may have developed to promote high quality and effective simulation-based education and training. For example, an Australian hospital has totally integrated translational simulation programmes as in-situ activities, not only as an educational approach but also more broadly to improve healthcare delivery and outcomes. This allows them to test systems and processes contextually, whilst also cautioning about potential associated risks related to staff, space, and equipment allocations among others [6].

In the UK, the expected impacts of simulation-based training on the healthcare educational system have resulted in new standards and guidelines for simulation practice that highlight the use of immersive technologies to support simulated learning [17–20]. These new guidelines require us to make some immediate changes in how we use and manage simulated practice. Immersive technologies can rely on hybrid forms of virtual simulation to recreate specific environments, situations, or even pathologies with no or limited reliance on physical elements. Virtual simulation is a broad term that refers to computer-based programs supported by different kinds of technologies that present authentic cases and scenarios mimicking real-life situations [21]. It includes, for example, screen-based serious games, computerised procedural simulators, virtual reality, extended and mixed reality, and augmented reality simulators [22, 23].

On the global level, the World Health Organisation Emergency Medical Teams 2030 Strategy [24] highlights that training and simulation exercises play an essential role in capacity building for emergency and disaster responses, with technology as one of the enablers. The North Atlantic Treaty Organization (NATO) Centre of Excellence for Military Medicine is focusing on emergency and disaster response capacity building, in line with a new strategy for effective joint training [25], and facilitates the world’s largest live simulation exercise, which happens every other year. This large-scale live simulation exercise engages 2,000 participants from 35 countries over two weeks. For countries with healthcare teams working in military or emergency disaster settings, the live simulation exercise supports capacity building by enhancing training in major trauma management and teamwork. Previous research results highlighted that virtual simulation plays an important part in preparedness of healthcare teams, especially for mixed multinational teams, in defining roles and responsibilities before attending the live simulation exercise, and also had a positive impact of their performance during the live simulation exercise [26].

Another example to consider is the collaborative work done between the main governmental healthcare institutions in Qatar, namely Primary Healthcare Corporation and Hamad Medical Corporation, its Ambulance Service, and the World Health Organisation (WHO) ahead of the International Federation of Football Associations (FIFA) World Cup Qatar 2022™. Among many other exercises that have taken place over several years before this major football tournament [27], they jointly organised a 4-h long system-wide tabletop exercise—guided by the WHO SimEX Manual and disseminated across multiple facilities—involving hundreds of participants [28, 29]. The simulated major incident was a hoax anthrax threat which created stampedes in multiple locations across the city and caused over 600 casualties. It was intended to test the major incident preparedness and resilience plan of all involved public health facilities, as well as the coordination capacity of the National Health Incident Command Centre (NHICC). Although the activity was officially scheduled—additional staff reported to work, in order not to disrupt normal patient care—the details of the scenario were unknown to the leadership and participants, to ensure that various aspects of the system could be thoroughly tested.

On the day of the tabletop exercise, all facilities and the Ambulance Service were activated in terms of manpower and resources as per their plan for a match day [28]. Such an approach, with this element of surprise, was not to deceive participants, but was done to test the system and help identify potential gaps in systems and processes [30]. The exercise was designed to also address specifically requested learning objectives by each facility, which meant that a very high number of learning objectives, which could be vastly different from one participating facility to another, could also be tested at a participant level as well as at a facility level. For example, the exercise team helped the Ambulance Service test and refine patient triaging, patient tracking and transportation capacity, and communication and coordination between the Emergency Services Dispatch Centre, the ambulance crews, and the many receiving facilities. Every participants’ actions were highly scrutinised by the concerned expert EPDR exercise evaluators allocated to each facility, as a form of formative assessment and to inform the production of the feedback report based on their observations.

At a smaller scale, a pilot study was conducted to test the effectiveness of an innovative training approach for paramedic students to develop casualty triage and management skills [31]. It involved online scenario-based Visually Enhanced Mental Simulation (VEMS) and a single-group pre- and post-intervention quasi-experimental design. From the study findings, online VEMS was determined to be an effective training modality which could help learners develop some disaster management and decision-making skills in relation to casualty triaging. This approach could be complementary to other training modalities already used to address these learning objectives [32]. Additionally, it has the advantage of being highly cost-effective, since it can be facilitated remotely with no physical resources other than a device with an internet connection, a communication platform with screen sharing capability, and an editable document so that scenario information such as text and images can be seen by all participants and observers. Although it relies on computer technology, it is more versatile and usable than a manikin representing a patient, or than some other physical simulator that can often only serve a limited purpose.

The success of these and many other EPDR simulations demonstrates a clear a need to share best practices and experiences regarding all forms of simulation-based system testing, training, and debriefing approaches in various contexts and to address different objectives. Together we can contribute to better capacity building in advancing interprofessional readiness for emergency preparedness and disaster responses. For this reason, we are inviting experts from various communities—military medical, healthcare, education, research, governmental, charitable and humanitarian relief agencies, and many others—to contribute to the special collection of Advances in Simulation with the theme “Simulation for Emergency Preparedness and Disaster Response”. We welcome reports and original research contributions on innovative work using virtual reality, screen-based simulation, or other simulation modalities, but also developmental work on interactive simulation-related applications supporting clinicians in decision-making in various disaster response situations. The scope of the type of simulated crisis situations that can be included in this special issue is broad, but should relate to health and care preparedness and disaster response training or system testing, for example to learn about human factors and teamwork in the context of a crisis situation or concerning crisis management and improving logistical aspects of disaster response (whether that be management of health care teams, equipment, resources, patient flow, relief efforts… or other key and important features of EDPR). Whether the work has already been applied or is still under development, we seek to create a forum sharing knowledge and experiences about training multi- or single agency teams to deliver care in high-risk environments and conflict zones, to deal with mass casualty incidents, and to prepare for large scale emergency events whilst keeping healthcare professionals safe and providing care for the communities we serve. We envision that this special collection will be about cross-learning and will create opportunities for new collaborations. “Combining forces” can only help to improve and enhance simulation-based practices for teams working in emergency preparedness and disaster response.

## Data Availability

Data sharing is not applicable to this article as no datasets were generated or analyzed during the current study.
